# Bacterial vaginal infections in diabetic and non-diabetic women

**DOI:** 10.1186/1471-2334-12-S1-P63

**Published:** 2012-05-04

**Authors:** K Lakshmi, Chitralekha Saikumar, V Illamani, Godfred A Menezes

**Affiliations:** 1 Department of Microbiology, Sree Balaji Medical College and Hospital (SBMCH), Chennai, India

## Background

Vaginal infection is a common disease of women. After 40 years, there is a fall in estrogen production. An estrogen deficient vagina as well as the immunocompromised status due to diabetes can lead to growth of abnormal flora which may in turn lead to infections. Bacterial vaginal infections are often least understood and empirical antifungal therapy for any vaginal infection without high vaginal swab culture is still in practice. The aim of the study is to analyze the prevalence of bacterial vaginal infections in diabetic and non-diabetic women.

## Methods

Fifty diabetic and fifty non-diabetic women of age 40-70 years were randomly selected from the patients attending SBMCH, Chennai. High vaginal swab specimens were collected from them and cultured aerobically and anaerobically. Biochemical tests were performed and the microorganisms identified. Antibiotic susceptibility pattern noted.

## Results

The microorganisms isolated were bacteria, *Candida spp*, *Trichomonas spp*. The major pathogens were *Escherichia coli* (15%), *Klebsiella pneumoniae* (2%), *Staphylococcus aureus* (9%) and *Candida* (16%). *Lactobacilli*, *Bacteroides fragilis* and *Peptostreptococcus spp*. were the anaerobes isolated. *E. coli*, *S.aureus*, *Candida spp*. were 18%, 12%, 18% reported in diabetic women and 12%, 6%, 14% reported in non-diabetic women respectively.

## Conclusion

The prevalence of pathogenic bacteria and Candida is more in diabetic women than the non-diabetic women. Pathogenic bacteria are found as frequently as the Candida. Hence, the practice of empirical antifungal therapy without taking high vaginal swab needs to be revised. The use of appropriate antibiotics along with antifungal drugs may be beneficial.

